# Quantitative Comparison of Hand Kinematics Measured with a Markerless Commercial Head-Mounted Display and a Marker-Based Motion Capture System in Stroke Survivors

**DOI:** 10.3390/s23187906

**Published:** 2023-09-15

**Authors:** Antonino Casile, Giulia Fregna, Vittorio Boarini, Chiara Paoluzzi, Fabio Manfredini, Nicola Lamberti, Andrea Baroni, Sofia Straudi

**Affiliations:** 1 Department of Biomedical and Dental Sciences and Morphofunctional Imaging, University of Messina, 98122 Messina, Italy; 2 Center of Translational Neurophysiology of Speech and Communication (CTNSC), Istituto Italiano di Tecnologia (IIT), 44121 Ferrara, Italy; boarinivittorio@gmail.com; 3 Doctoral Program in Translational Neurosciences and Neurotechnologies, University of Ferrara, 44121 Ferrara, Italy; giulia.fregna@unife.it; 4 Department of Mathematics and Computer Science, University of Ferrara, 44121 Ferrara, Italy; 5 Department of Neuroscience and Rehabilitation, University of Ferrara, 44121 Ferrara, Italy; chiara.paoluzzi@edu.unife.it (C.P.); nicola.lamberti@unife.it (N.L.); brnndr3@unife.it (A.B.); strsfo@unife.it (S.S.); 6 Department of Neuroscience, Ferrara University Hospital, 44124 Ferrara, Italy

**Keywords:** markerless motion capture system, head-mounted display, stroke, motion analysis, motor recovery, Oculus Quest, Optitrack

## Abstract

Upper-limb paresis is common after stroke. An important tool to assess motor recovery is to use marker-based motion capture systems to measure the kinematic characteristics of patients’ movements in ecological scenarios. These systems are, however, very expensive and not readily available for many rehabilitation units. Here, we explored whether the markerless hand motion capabilities of the cost-effective Oculus Quest head-mounted display could be used to provide clinically meaningful measures. A total of 14 stroke patients executed ecologically relevant upper-limb tasks in an immersive virtual environment. During task execution, we recorded their hand movements simultaneously by means of the Oculus Quest’s and a marker-based motion capture system. Our results showed that the markerless estimates of the hand position and peak velocity provided by the Oculus Quest were in very close agreement with those provided by a marker-based commercial system with their regression line having a slope close to 1 (maximum distance: mean slope = 0.94 ± 0.1; peak velocity: mean slope = 1.06 ± 0.12). Furthermore, the Oculus Quest had virtually the same sensitivity as that of a commercial system in distinguishing healthy from pathological kinematic measures. The Oculus Quest was as accurate as a commercial marker-based system in measuring clinically meaningful upper-limb kinematic parameters in stroke patients.

## 1. Introduction

Stroke is one of the main causes of acquired adult disability worldwide [1,2,3]. Even worse, due to the ongoing ageing of the population, the impact of stroke is expected to dramatically increase in the coming years [4]. In addition to obvious human costs, stroke is also associated with very high direct and indirect economic costs [5] that, in 2017, were estimated at EUR 60 billion across 32 European countries [6]. Upper-limb impairment is one of the most frequent and disabling consequences after stroke, persisting at 6 months in approximately 60% of people [7]. Rehabilitation has a key role in maximizing upper-limb motor recovery, neuroplasticity can be elicited through motor and cognitive stimulations in order to improve brain re-organization and neuromotor abilities [8]. Physical-therapy-based rehabilitation has, in fact, showed notable effects in improving upper-limb function, increasing muscle strength, reducing arm pain, and increasing quality of life [9]. Upper-limb movements are instrumental to perform daily living routines (e.g., pouring a glass of water or eating) that are critical for the quality of life of stroke survivors [10,11]. Their preservation/re-establishment is, thus, one of the key goals of motor rehabilitation therapies.

A key step towards providing effective upper-limb-rehabilitation interventions for post-stroke patients is to establish sensitive and reliable assessments of motor functions. Kinematic analysis is presently one of the most sophisticated techniques in the clinician’s toolbox to quantitatively investigate motor performance, to assess the quality of arm movements and to detect behavioral changes during functional recovery and rehabilitation [12]. Clinicians have developed several kinematic metrics of upper-limb movements to characterize the amount of impairment and monitor patient’s sensorimotor recovery in a reliable way [13]. Among these, hand peak velocity represents a relevant indicator of motor changes in stroke subjects [14] with good reliability properties [13]. Moreover, in kinematic evaluation, hand movement trajectories are frequently recorded for computing movement accuracy metrics [13], useful data for analyzing upper-limb function in stroke survivors. Given the high heterogeneity reported in the literature concerning the technology used, motor tasks performed, and kinematic metrics analyzed [15], specific recommendations have been made to standardize the methodologies to assess upper-limb kinematics after stroke [16]. For example, high-speed and high-resolution marker-based systems are recommended for kinematic analysis. These systems have, however, several limitations in terms of applicability due to high costs, the need of a dedicated area, trained professionals requested, and non-trivial set-up procedures that are beyond the motor capabilities of patients with neurological diseases [17]. To overcome these limitations, several commercial markerless devices, such as Kinect or Leap Motion Controller, have been adapted to the clinical use [18]. These markerless tracking devices hold the promises to allow clinicians to perform kinematic assessments in their everyday clinical practice at a fraction of the costs of optical systems and under more ecological conditions (e.g., no marker needs to be placed on the patient). Assessment through markerless technologies has been mostly investigated in the context of gait analysis [17,19], with some studies addressing the analysis of upper-limb movements [20,21,22], but rarely in post-stroke patients [23].

The interest of clinicians for markerless tracking devices is currently increasing with a specific focus on head-mounted displays (HMDs). An HMD is a device that is worn on the head and binocularly displays a virtual environment (henceforth VR) in an immersive manner. VR is being widely used in the scientific community as a research tool. For example, several studies have used embodiment in virtual avatars as a manner to investigate human cognition or explore new ways to treat psychological conditions [24,25,26,27,28,29,30]. Notably, many presently available HMDs, such as the Oculus Quest (Meta, Menlo Park, CA, USA) or the Vive (HTC, Xindian, New Taipei, Taiwan) also have motion-tracking capabilities to monitor, in real time, the movements. They, thus, represent a promising solution for combining a highly stimulating intervention in an enriched environment with a portable and flexible motion capture system. Given the characteristics of modern HMDs, there are presently several ongoing studies to use them in the rehabilitation of neurological disorders such as multiple sclerosis [31] and stroke [32]. However, while the clinical outcomes of immersive VR-based rehabilitation interventions have been thoroughly investigated [32,33,34], their potential use as kinematic measurement devices has receive significant less attention. In particular, very few studies have analyzed the kinematic accuracy of HMDs compared to marker-based systems [35,36,37,38] and no study has investigated HMDs’ hand-tacking precision in post-stroke subjects.

We recently developed an immersive virtual reality tool (henceforth RehabVR) for upper-limb rehabilitation based on the Oculus Quest 2 HMD [39]. We performed a preliminary study to test feasibility, safety, and acceptance in stroke patients who completed a single session of upper-limb training in our immersive environment. Our findings showed not only very high levels of satisfaction and embodiment in all patients of our cohort but also revealed correlations between behavioral measures of patients’ performance (i.e., median difference in completion time between the paretic and non-paretic arm) and clinical scores (i.e., the Fugl–Meyer Assessment score). This result suggested that behavioral measures, which are easily computed by our immersive VR-based tool, could be potentially used as proxies of clinical assessments that need instead long testing times and specifically trained personnel.

The present study aimed to explore whether, in addition to behavioral measures, the measures of hand kinematics provided by the Quest 2 could be potentially used for clinical purposes. This is the first study that tested HMD accuracy in motion tracking measurement in post-stroke patients, potentially suggesting new ways to remotely monitor upper-limb recovery through kinematic data. To this end, we recorded the hand trajectories of post-stroke patients, while they executed one of the tasks in RehabVR, simultaneously by means of the Quest 2 and a commercial marker-based motion capture system (Optitrack by Natural Point Inc., Corballis, OR, USA). We then compared the same kinematic assessments computed separately from each of the two data sets.

## 2. Materials and Methods

### 2.1. Subjects

A total of 14 subacute and chronic post-stroke patients (4 females, mean age 59 ± 15) enrolled from the Rehabilitation Units of the Ferrara University Hospital participated in the experiments. They had a wide range of motor impairments and a diagnosis of first, ischemic, or hemorrhagic stroke. No age restrictions were applied but patients affected by severe cognitive impairments or other severe co-existing clinical conditions were excluded. The clinical protocol and all procedures were approved by the local ethical committee (Comitato Etico di Area Vasta Emilia Centro (CE-AVEC) protocol code: 897-2020-Oss-AOUFe approved on 17 March 2021).

### 2.2. Experimental Procedures

**Figure 1:**
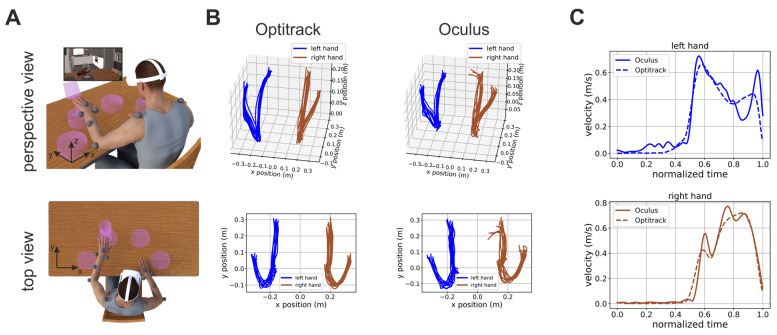
Experimental protocol and examples of hand trajectories and velocity profiles. (**A**) Perspective (top panel) and top (bottom panel) view of a graphical depiction of the experimental conditions. The patients wore a head-mounted display and were immersed in a virtual environment in which they played a game with motor rehabilitation purposes (i.e., hit virtual glasses and make them fall). During the task, their hand movements were recorded by the Oculus on-board sensors and software and by means of a commercial motion capture system (Optitrack, NaturalPoint, Inc., Corvallis, OR, USA), respectively. The gray circles represent the placements on the patient’s body of the Optitrack markers. The inset in the top panel shows the patient’s view of the virtual environment inside the HMD. (**B**) Perspective (top panel) and top (bottom panel) view of hand trajectories recorded by the Optitrack and Oculus systems during one experimental session. Left and right hand trajectories are plotted in blue and light brown, respectively. (**C**) Examples of speed profiles computed from Oculus (solid line) and Optitrack (dashed line) data for the left (top panel) and right (bottom panel) hand, respectively. Further examples of hand speed profiles are shown in Figure S1.

Prior to the experimental procedure, written, informed consent was obtained from all patients. After being consented, patients sat in front of a table and a total of 13 reflective markers were applied on their arms and shoulders (see markers’ placements in Figure 1A). Following that, the patient was comfortably seated in front of a table and wore a head-mounted display (HMD, Oculus Quest 2, Meta, USA) and she/he was immersed in an in-home developed immersive virtual environment for motor rehabilitation [39].

The system offers different motor rehabilitation tasks and, for the purpose of this study, we focused on the “*Glasses*” task, since it affords the reaching movements requested in upper-limb kinematics examination in post-stroke patients [16].

In this task, the patient was presented with four pedestals placed on a virtual table in front of her/him. The pedestals were distributed along a circle centered on the patient’s body at equal angular displacements (Figure 1A). A trial start when the patient placed her/his hands on two locations marked on the virtual table. A glass then appeared on one randomly selected pedestal and the patients had to push it down (Figure 1A). The patients had to use the hand closer to the pedestal on which the glass appeared (two pedestals were closer to the right hand and two were closer to the left hand).

For the purpose of this study, the patients performed three sessions of the “Glasses” task, each consisting of 40 trials: 20 trials for each hand.

### 2.3. Motion Capture

During task execution, we recorded the patients’ hand positions by means of the Oculus and of a commercial motion capture system (Optitrack, Natural Point Inc., Corballis, OR, USA) equipped with 6 cameras. The Oculus estimates hand positions and postures by a combination of on-board cameras and software. The Oculus is equipped with 4 onboard cameras placed at the corners of the HMD that are used to capture images of the subjects’ hands during task performance. The software running on the Oculus uses computer vision routines to segment the images of the hands from the background and to estimate their posture. The spatial location of the hands is computed by exploiting stereoscopic depth information provided by different cameras. The specific details of this process are patented and they are, thus, not publicly available.

The Optitrack system uses a set of infrared cameras to track, by triangulation, the positions of a set of infrared-reflective markers.

To allow synchronization between the data recorded on the Oculus and Optitrack systems, this latter system broadcasted, in real time, the unique identifier of each acquired frame on the local network. This stream of data was received by our VR system on the Oculus Quest and was used to timestamp the locally recorded hand positions, together with the time instants were each trial of the task started and ended. Optitrack data were off-line processed to interpolate gaps in the markers’ position and then exported for further processing.

In the Optitrack system, we computed the position of the left and right hand by averaging the positions of the two markers placed on each of the two wrists. In the Oculus system, we directly used the hand positions computed by onboard software with no further processing.

### 2.4. Data Analysis

To remove noise, we first low-pass filtered Optitrack and Oculus hand positions at 3 Hz by means of a 2nd order Butterworth filter. We then segmented both the Optitrack and Oculus data into trials by using the timestamped events recorded by our VR system during task execution. For each trial, we used the Optitrack data to obtain the time t_max_ at which the hand performing the task was maximally extended with respect to its initial position at the beginning of the trial (time t = 0). We then extracted the hand position between 0 and t_max_ both from the Optitrack and Oculus data and computed the maximum distance travelled by the hand performing the task. We then computed the peak velocity of the hand in the interval [0, t_max_] both from Oculus and Optitrack data.

The Oculus system estimates the position of the two hands by means of onboard software and cameras. Such tracking can be sometime faulty (e.g., because of abrupt movements of the head, where the sensors are located, or sudden changes of direction of the hand, etc.). In these cases, the Oculus will incorrectly locate the hand position, which will default to a pre-defined value (e.g., 0,0,0) and a consequent “spike” in the velocity profile. To discard such incorrect data from our analysis, we excluded all trials in which the maximum distance and peak velocity computed from Oculus data were above a threshold of 65 cm and 2 m/s, respectively. These higher bounds were set based on the ground-truth values provided by the Optitrack system that yielded a maximum distance of 51 cm and a maximum peak velocity of 1.35 m/s across all subjects and trials.

All pre-processing steps were performed using Python scripts.

### 2.5. Analysis of the Potential Spatial Dependence of Measurement Errors

To investigate whether the errors in estimating the patient’s hands position were uniform or not across the workspace, we used the percentages of rejected trial as a proxy measure of accuracy and submitted them to a one-way ANOVA with factor the spatial locations of the targets (i.e., the four purple pedestals places as to uniformly span the workspace from left to right; see Figure 1).

### 2.6. Linear Regression Analysis

To investigate the congruency between Optritrack and Oculus measures, we performed a linear correlation analysis followed by an ANOVA on the values of the slope. All statistical analyses were performed in R.

## 3. Results

In total, 14 post-stroke subjects were included in the present study, and the demographic and clinical characteristics of the sample are reported in Table 1. Figure 1B shows examples of hand trajectories recorded by the Optitrack (left column) and Oculus (right column) systems. Although the Oculus Quest trajectories were, as expected, noisier, they were, nonetheless, very close to their ground-truth values. The similarity of Oculus estimate to their ground-truth values was also true for estimates of hand velocity, although, in this case, higher-frequency noise was present (Figure 1C and Figure S1). This was expected. Indeed, the velocity is the first derivative of position, and the derivative operator magnifies higher frequency noise.

### 3.1. Oculus Estimates of Position and Velocity Are Linearly Related to Their Ground-Truth Values

**Figure 2:**
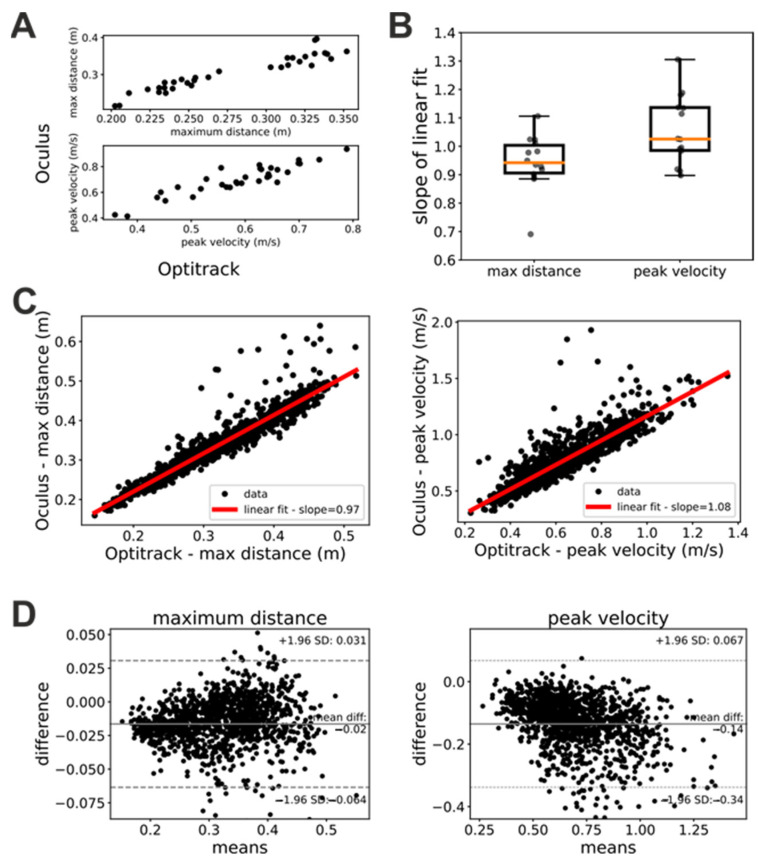
Estimation of distances and velocities by the Optitrack and Oculus systems, respectively. (**A**) Maximum reaching distances (top) and peak velocities (bottom) measured in an experimental session for one of our patients. In both panels, each dot represents a single trial. (**B**) Slopes of the linear regression between measures of maximum reaching distance and peal velocity computed from Optitrack and Oculus data. Each dot represents a patient. Mean slope for estimates of maximum reaching distance = 0.94 ± 0.1. Mean slope for estimates of peak velocities = 1.06 ± 0.12. (**C**) Estimates of maximum reaching distances (left panel) and peak velocity (right panel) computed for all patients from Optitrack and Oculus data. Each dot represents a single trial. In both panels, the red line represents a linear fit of the data. The slopes of this fit is shown in the caption. (**D**) Bland–Altman plots of the data in panel (**C**).

To quantitatively investigate the hand tracking capabilities of the Oculus system, we computed for each trial the maximum reaching distance and peak velocity of the patients’ hands and we compared these estimates with their ground-truth values provided by the Optitrack system. Figure 2A shows scatterplots of measures of maximum hand distance and velocity provided by the Oculus and Optitrack systems, respectively. In these plots, each dot represents a single trial whose x coordinate represents the measure provided by the Optitrack and whose y coordinate represents the measure provided by the Oculus system. As these plots show, the Oculus and Optitrack measures exhibited a clear linear relationship, and thus, we investigated the slope of this relationship.

In a first step, we studied whether this linear relationship was stable in time (i.e., across the three sessions of the “Glasses” task) and independent from the hand. To this end, we performed linear fits of the Oculus against Optitrack measures of maximum distance and peak velocity separately for each patient, trial, and acting hand. We then submitted the slopes of these linear fits to a repeated-measures ANOVA analysis with factors hand (left or right) and session (first, second, or third). Both factors as well as their interaction were not significant for both the maximum distance (factor session: F(2, 26) = 0.76, *p* = 0.48; factor hand: F(1, 13) = 0.155, *p* = 0.7; interaction: F(2, 26) = 0.71, *p* = 0.5) and peak velocity (factor session: F(2, 26) = 1.09, *p* = 0.35; factor hand: F(1, 13) = 0.183, *p* = 0.68; interaction: F(2, 26) = 0.332, *p* = 0.72) ANOVA. A similar pattern of result was obtained when trials were sorted based on whether they were executed by patients with their healthy or impaired hand (ANOVA maximum distance: factor session: F(2, 26) = 0.76, *p* = 0.48; factor hand: F(1, 13) = 0.58, *p* = 0.46; interaction: F(2, 26) = 0.9, *p* = 0.42 – ANOVA peak velocity: factor session: F(2, 26) = 1.90, *p* = 0.35; factor hand: F(1, 13) = 0.21, *p* = 0.65; interaction: F(2, 26) = 1.45, *p* = 0.25). These results show that the linear relationship between estimates provided by the Oculus system and their ground-truth values were robust in time and had similar characteristics for the two hands.

Since both the factor session and hand were not significant, we pooled together the data for each patient and computed the slope of the linear relationship between Oculus and Optitrack kinematic measure. Figure 2B shows the distribution of the slopes across patients. For both maximum distance and peak velocity, the average slope was very close to 1 (maximum distance: mean slope = 0.94 ± 0.1; peak velocity: mean slope = 1.06 ± 0.12). We obtained similar results when we pooled together the data from all participants, sessions, and trials (Figure 2C). In this case, we obtained a mean slope of 0.97 for the measures of maximum distance and of 1.08 for the measures of peak velocity. The Bland–Altman plots (Figure 2D) showed a systematic bias both for measures of maximum distance (bias = 0.01 m) and peak velocity (bias = −0.14 m/s). A negative trend was present in both Bland–Altman plots. It was just noticeable in the maximum distance plot (Figure 2D, left panel), and very evident in the peak velocity plot (Figure 2D, right panel).

Together with our ANOVA analysis above, the results in Figure 2 suggest that, provided that some simple data-cleaning procedures are enforced (see Section 2), the kinematic measures of distance and velocity provided by the Oculus are relatively accurate and linearly related to their ground-truth values. The slopes of these linear relationships suggest that the Oculus system tends to slightly underestimate distances and to slightly overestimate velocities. Furthermore, the negative trends in the Bland–Altman plots suggest that in both distance and velocity estimates of the Oculus system, there is a proportional bias that is more pronounced for velocity measures.

### 3.2. Oculus Kinematic Assessments Agree with the Same Assessments Based on Ground-Truth Data

**Figure 3:**
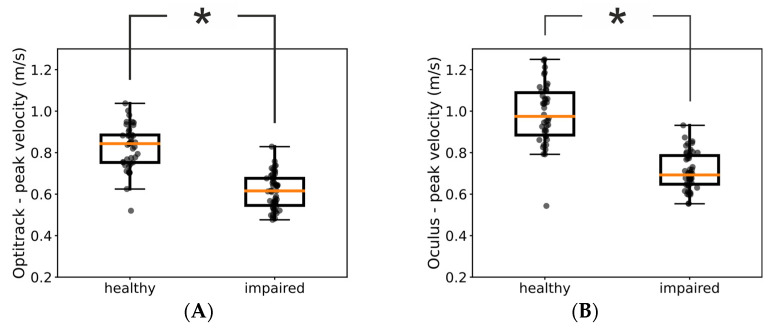
Comparison of kinematic assessments using data from the Optitrack and Oculus systems. The two boxplots show the distributions of peak velocities of healthy and impaired hand obtained with the Optitrack (**A**) and Oculus (**B**) systems in one patient. In both panels, each dot represents a trial and the asterisk indicates a statistically significant difference (*p* < 0.05).

We next investigated whether kinematic measures provided by the Oculus were sensitive enough to reveal fine-structured characteristics of the patients’ movements. For example, Figure 3A shows the distributions of peak velocities measured by means of the Optitrack system in one of our patients for the impaired and healthy hand, respectively. For this patient, the medians of the two distributions were significantly different (Mann–Whitney U test = 126, *p* << 0.05) and we found the same significant difference between the impaired and healthy hand also in the Oculus data (Figure 3B, Mann–Whitney U test = 107, *p* << 0.05).

Thus, we investigated the congruency between assessments based on ground-truth Optitrack data and Oculus data across our pool of patients. When we used the Optitrack estimates of peak velocities, we found a significant difference between the distributions of the healthy and impaired hand in 11 out of 14 of our patients. Notably, for 10 of them, we found the same significant difference also in the peak velocities assessed by means of the Oculus system (Table 2). These results further suggest that kinematic assessments obtained with the Oculus system can be used as proxy for the same assessments obtained by means of ground-truth values.

A number of trials were excluded from our analysis because they contained unnatural “spikes” of velocity due to the unavoidable errors produced by the Oculus in estimating the position of the patient’s hands. A question arises as to whether the magnitude of these errors was uniform or not across the workspace. To investigate this point, we used the percentage of rejected trials as a proxy measure of the accuracy of the Oculus Quest. A one-way ANOVA analysis revealed that this percentage was not modulated by the spatial position of the target object and, thus, of the trajectory of the patient’s hand (F(3, 39) = 2.29, *p* = 0.09). Although, further and more accurate measures are needed, this result seems to suggest that the estimation performance of the Oculus Quest 2 were homogeneous across the workspace.

## 4. Discussion

Here, we quantitatively compared the accuracy of the Oculus Quest 2 in tracking the hand movements of a group of stroke patients with that of a commercial marker-based system (Figure 1). Our results showed that the estimates of the hand position and peak velocity provided by the markerless Oculus Quest were in very close agreement with those provided by a marker-based commercial system. Indeed, the two sets of measures were not only very strongly correlated but the regression line between them exhibited a slope close to 1 (Figure 2). Furthermore, the Oculus Quest exhibited a sensitivity very similar to that of the commercial system in distinguishing pathological from healthy upper-limb movements (Figure 3 and Table 2).

Especially in patients with neurological disorders, such as stroke, a quantitative analysis of the subject’s motor performance is crucial to plan proper therapeutic interventions. Marker-based motion capture systems represent the reference standard for clinical kinematic assessments [16] but have high costs and need lengthy training periods. As such, there is a growing interest by clinicians towards more cost-effective and easy-to-use markerless systems. In particular, the COVID-19 outbreak has underlined the clinical need to have systems able to assess patient’s physical function more flexibly. The research of new clinical tools for the assessment of upper-limb function in post-stroke patients at home is, thus, increasing. Among them, wearable technologies (like Inertial Measurement Units sensors) are under investigation [40,41] and also video-conferencing administration of validated clinical scales [42], or ad hoc developed tools have been recently proposed [43]. However, the related literature is still lacking and there is a paucity of technologically and clinically validated solutions for remote kinematic analyses in subjects with neurological disorders.

While several studies showed promising results in upper-limb kinematic assessment in post-stroke people through markerless devices [44], there is scarce information on validation data and psychometric properties of kinematic metrics recorded with these tools [45]. Among the studies that simultaneously recorded and compared upper-limb motor indexes by means of markerless (i.e., Kinect, Leap Motion Controller) and marker-based systems in post-stroke subjects, Bonnechère et al. found good agreement in speed-related parameters among Kinect sensor and PiG (Vicon) system in 10 chronic stroke patients analyzed on shoulder and wrist movements [23]. However, the small sample size influences the strength of the results. Furthermore, Bonnechere et al. focused only on shoulder movements and performed no measures of hand kinematics.

The clinical applications of HMDs are currently increasing due to their affordable costs and ease of use. Concerning the proofs of accuracy in kinematic assessment made so far, HMDs showed encouraging results both in simulated analysis [35] and in human testing. Good estimations have been detected in assessing cervical spine mobility [37], balance stability [36], and shoulder range of motion [38] compared to reference marker-based systems; however, only healthy participants have been involved. Motor performance in patients after stroke is definitely different compared to what is observed in the general population and no one has still investigated HMDs for motor recording analysis in this patient population, specifically in hand kinematics assessment.

“Reaching a glass” is one of the recommended functional tasks for the evaluation of upper-limb functions [16]. Indeed, the characteristic of these movements, such as the peak velocity, can provide relevant information to the clinician on the participant’s level of impairment [46,47]. Our implementation of the “reaching a glass” task in RehabVR contained the reaching phase but lacked the grasping phase because of the present impossibility to physically interact with objects in a virtual environment. However, the reaching phase alone already offers several informative data on upper-limb motor functions [46]. Furthermore, the rapid development of haptic interfaces holds the promise to remove this limitation in the close future.

Our study also had limitations that need to be discussed. First, the generalizability of our results was influenced by the reduced sample size. Second, the motion capture capabilities of the Oculus Quest were presently limited to hand movements. Kinematic analysis of the hands alone does not allow a comprehensive evaluation of the patient’s upper-limb functions. For example, it does not allow to evaluate potential compensatory movements with other joints (e.g., the shoulder) or motor synergies. However, the possibility, suggested here, of measuring hand movements by means of an easy-to-use and cost-effective system such as the Oculus Quest 2 paves the way for a new generation of systems that can provide quick and reliable proxy assessments of patients’ improvements. Notably, these assessments could be potentially performed remotely, thus strongly reducing the number of in-person and more cost-demanding in-depth evaluations. Third, our analysis showed that while the position information provided by the Oculus Quest 2 are fairly accurate, its velocity estimates were affected by a higher level of noise and acceleration estimates are unreliable (data not reported here). While this limits the possibility of using the Oculus Quest 2 to compute kinematic assessments that are based on the acceleration and higher derivatives (e.g., measures based on the smoothness of the movement [13]), it might also stimulate research into finding novel measures that are based on position and velocity alone. Such measures would have a wide use in the clinical use, given that they could be easily performed with the new cost-effective devices available today. In addition, RehabVR could be augmented with a set of inertial sensors placed on the patient’s body. Such sensors have a low cost and could provide estimates of acceleration to complement the position and velocity measures provided by the Oculus Quest.

## 5. Conclusions

In a cohort of 14 stroke patients (FMA-UE range 20–65), the Oculus Quest 2 exhibited an accuracy similar to that of a commercial marker-based system in measuring two clinically relevant kinematic parameters of upper-limb movements: the spatial ranges of hand movements and their peak velocities. These results suggest that the markerless motion capture capabilities of the Oculus Quest can be used to monitor arm motor recovery in stroke patients.

## Figures and Tables

**Table 1:** Demographic and clinical characteristics of the patients enrolled, expressed in relative and absolute frequencies (n, %). Abbreviations: SD: standard deviation, FMA-UE: Fugl–Meyer Assessment-Upper Extremity.

	Sample (N = 14)
**Age** (mean, SD)	59 ± 15
Sex	
Males	10 (71%)
Females	4 (29%)
**Stroke Type**	
Ischemic	13 (93%)
Hemorrhagic	1 (7%)
**Stroke side**	
Left	5 (36%)
Right	9 (64%)
**Stroke timeframe**	
<1 year	6 (42%)
1–3 years	4 (29%)
>3 years	4 (29%)
**FMA-UE score** (min-max)	20–65

**Table 2:** Statistical comparison of the peak-velocity distributions for the healthy and impaired hand. The top row represents p-values obtained using ground-truth Optitrack values and the bottom row *p*-values obtained using Oculus measures. Each column represents results for a patient. For display purposes significant values at the *p* < 0.05 level are highlighted in red.

	1	2	3	4	5	6	7	8	9	10	11	12	13	14
Optitrack	7.37 × 10^−3^	8.23 × 10^−4^	1.80 × 10^−4^	5.77 × 10^−15^	6.36 × 10^−1^	4.44 × 10^−3^	1.81 × 10^−3^	2.36 × 10^−1^	4.32 × 10^−8^	2.40 × 10^−5^	3.06 × 10^−13^	2.15 × 10^−10^	2.60 × 10^−1^	1.10 × 10^−4^
Oculus	5.35 × 10^−1^	7.02 × 10^−3^	3.69 × 10^−2^	8.24 × 10^−13^	2.84 × 10^−1^	1.27 × 10^−3^	8.73 × 10^−4^	1.44 × 10^−1^	1.16 × 10^−6^	1.12 × 10^−2^	1.02 × 10^−13^	1.02 × 10^−5^	1.43 × 10^−1^	4.56 × 10^−4^

## Data Availability

The data that support the findings of this study are available from the corresponding author, upon reasonable request.

## Supplementary Materials

The following supporting information can be downloaded at: https://www.mdpi.com/article/10.3390/s23187906/s1, Figure S1: Examples of speed profiles computed from Oculus (solid line) and Optitrack (dashed line) data for the left (left column) and right (right column) hand, respectively for four different patients.
