# Supplementary material for: Quantitative Comparison of Hand Kinematics Measured with a Markerless Commercial Head-Mounted Display and a Marker-Based Motion Capture System in Stroke Survivors
Source: Sensors (Basel). 2023 Sep 15;23(18):7906. doi: 10.3390/s23187906 (PMC10535006; doi:10.3390/s23187906)
Supplement: Supplementary file 1 [file sensors-23-07906-s001.zip › sensors-2542825-supplementary.pdf]

Supplementary Material

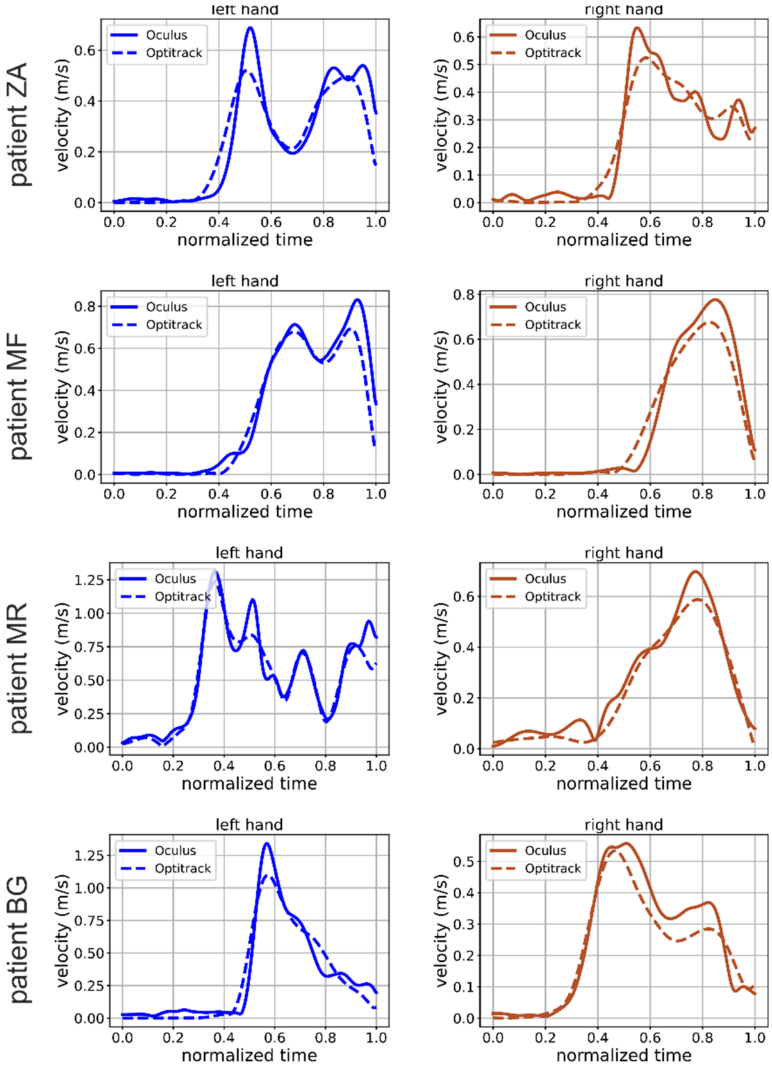

Figure S1. Examples of speed profiles computed from Oculus (solid line) and Optitrack (dashed line) data for the left (left column) and right (right column) hand, respectively for four different patients.
